# Alterations of Antioxidant Enzymes and Biomarkers of Nitro-oxidative Stress in Tissues of Bladder Cancer

**DOI:** 10.1155/2019/2730896

**Published:** 2019-05-05

**Authors:** Md Obaidul Islam, Tiziana Bacchetti, Gianna Ferretti

**Affiliations:** Department of Life and Environmental Sciences (DiSVA), Department of Clinical Science and Odontostomatology (DISCO), Polytechnic University of Marche, Ancona, Italy

## Abstract

Bladder cancer (BC) is one of the most common tumors found in the urinary bladder for both male and female in western countries. In vitro and in vivo studies suggest that high levels of reactive oxygen species (ROS) and reactive nitrogen species (RNS) and oxidative stress play a crucial role in human cancer. Low concentration of ROS and RNS is indispensable for cell survival and proliferation. However, high concentration of ROS and RNS can exert a cytotoxic effect. Increased oxidative stress is a result of either increased ROS/RNS production or a decrease of antioxidant defense mechanisms. A literature search was carried out on PubMed, Medline, and Google Scholar for articles in English published up to May 2018 using the following keywords: oxidative stress, antioxidants, reactive oxygen species, lipid peroxidation, paraoxonase, urinary bladder cancer, and nitric oxide. Literature data demonstrate that BC is associated with oxidative stress and with an imbalance between oxidants and antioxidant enzymes. Markers of lipid peroxidation, protein and nucleic acid oxidation are significantly higher in tissues of patients with BC compared with control groups. A decrease of activity of antioxidant enzymes (superoxide dismutase, catalase, glutathione, and paraoxonase) has also been demonstrated. The imbalance between oxidants and antioxidants could have a potential role in the etiology and progression of bladder cancer.

## 1. Introduction

Bladder cancer (BC), the most common malignancy of the urinary tract, represents a crucial public health hazard due to its high aggressiveness and poor prognosis [1]. BC is the 3rd most common cancer in men and 11th most common cancer in women. Transitional bladder tumors originating from urothelial cells are classified as nonmuscle invasive (NMI) (pTa or pT1) or invasive (pT2, pT3, or pT4) with the latter carrying a worse prognosis. BC has the highest recurrence rate of any other solid tumors, in which most of them exceed relapses or progresses from nonmuscle invasive to muscle invasive disease. Newly diagnosed patients, approximately 70% to 80%, have non-muscle invasive tumors and are managed by combined therapy with transurethral resection or radical cystectomy and intravesical chemotherapy [2, 3].

**Figure 1:**
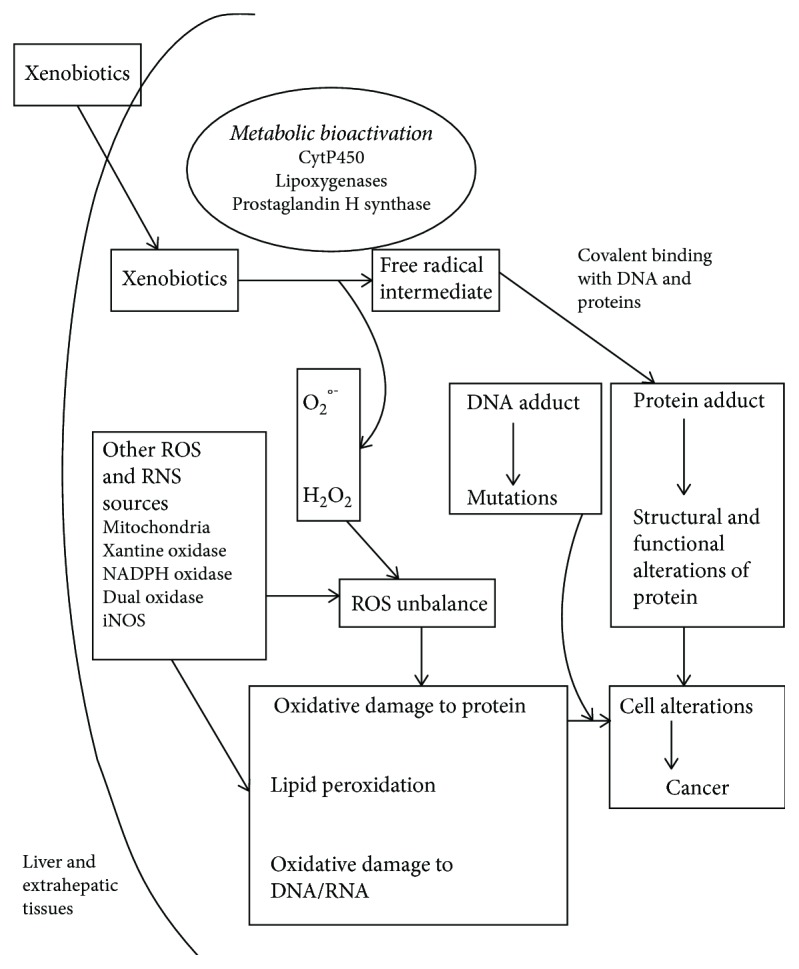
Factors potentially involved in oxidative stress in bladder cancer. Formation of xenobiotic free radical intermediates during biochemical pathways catalyzed by cytochrome P450, lipoxygenase, prostaglandin H synthase, and cellular effects of reactive oxygen species (ROS) and reactive nitrogen species (RNS).

Genetic and environmental factors have been implicated in BC etiology as recently reviewed [4, 5]. Dietary factors such as arsenic and/or environmental xenobiotics can be metabolized in the human body, and carcinogenic byproducts reach the urinary bladder via urinary excretion [5, 6]. The molecular mechanisms by which metalloids, smoking and xenobiotics are correlated with increased oxidative stress and are potentially involved in BC have been previously studied [6–9]. Trivalent inorganic arsenic inhibits cell enzymes by binding to the sulfhydryl groups of dihydrolipoamide, resulting in a decreased production of cellular ATP. Moreover, trivalent arsenic inhibits the production of glutathione which protects cells against reactive oxygen species (ROS). Many other mechanistic studies of arsenic toxicity involve reactive oxygen species and reactive nitrogen species (RNS) generated during inorganic arsenic metabolism in living cells [6]. Tobacco smoke contains many carcinogens like polycyclic aromatic hydrocarbons (PAHs) and nitrosamines. Cigarette smoking increases the formation of ROS and RNS and together results in nitration and oxidation of plasma proteins [7, 8]. The metabolic conversion of xenobiotic chemicals in liver and extrahepatic tissues represents a key element in understanding the effects and the toxicity of industrial/environmental chemicals. During metabolic bioactivation of xenobiotics, oxidation reactions mostly catalyzed by cytochrome P-450s can lead to reactive electrophilic compounds. Cytochrome P-450s can also act as a reducing system that leads to radical intermediates which may react on oxygen to produce many ROS leading to oxidative stress. Other enzymes such as prostaglandin H synthase (PHS) contribute to metabolic bioactivation in extrahepatic tissues [10]. PHS exerts a role in prostaglandin generating substrate-derived free radical intermediates which can oxidize xenobiotics to biologically reactive intermediates converting them to mutagenic and carcinogenic forms (Figure 1). Eling et al. [10] has also reported relatively high levels of PHS and low levels of cytochrome P-450 in bladder epithelium. Even lipoxygenases contribute to *in vivo* metabolism of xenobiotics in mammals [11] (Figure 1). This metabolism is due to two enzymatic activities, a cyclooxygenase and a peroxidase.

Intracellular antioxidant enzymes such as superoxide dismutase (SOD), catalase (CAT), and glutathione peroxidase (GTPx) exert a protective effect against oxidative damage [12]. Among detoxification and/or antioxidant systems, a key role is exerted also by the family of enzyme paraoxonases (PONs) whose physiopathological relevance in different human diseases associated with oxidative stress has been widely demonstrated [13]. Alterations of the balance of antioxidants/oxidants have a significant role in the pathogenesis of different kinds of tumors [12–16].

The literature review summarizes the state of our knowledge regarding alterations of ROS/RNS production, modifications of antioxidant enzymes, and markers of oxidative stress in bladder cancer as demonstrated in human subjects. To reach this objective, we performed a search on PubMed, Medline, and Google Scholar for articles in English published up to May 2018 using the following keywords: oxidative stress, antioxidants; catalase (CAT), superoxide dismutase (SOD), glutathione peroxidase (GTPx), reactive oxygen species (ROS), reactive nitrogen species (RNS), lipid peroxidation, paraoxonases (PONs), urinary bladder cancer, and nitric oxide.

## 2. Oxidative Stress: Role of Reactive Oxygen Species (ROS) and Reactive Nitrogen Species (RNS)

**Figure 2:**
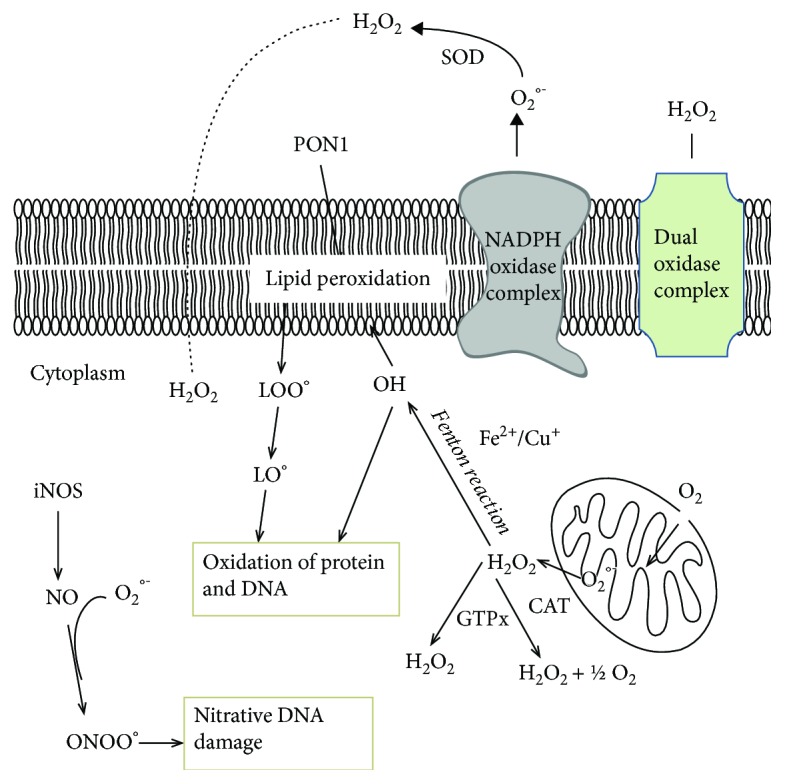
Formation of reactive oxygen species (ROS) and reactive nitrogen species (RNS) and antioxidant mechanism. Superoxide generated by NADPH oxidase complex and other different pathways may be degraded by cytosolic superoxide dismutase (SOD) and by the mitochondrial manganese-containing SOD (MnSOD) to H_2_O_2_. H_2_O_2_ is further eliminated by catalase (CAT) and glutathione peroxidase (GTPx) enzymes. Hydrogen peroxide is produced also by the dual oxidase complex (Duox). H_2_O_2_ is able to cross cell membranes, and within the cells, it can react with Cu^+^/Fe^2+^ to form hydroxyl radicals via Fenton reaction. Nitric oxide is generated from inducible nitric oxide (iNOS). From reaction between superoxides with nitric oxide, peroxynitrite (ONOO^−^) is formed. Peroxynitrite (ONOO^−^) can damage a wide array of molecules in cells, including DNA and proteins. Reactive oxygen species (ROS) may either react directly with some amino acid residues or lead to the oxidative cleavage of the protein backbone. Other possible formation routes of protein oxidation are via the oxidation of lipids resulting in reactive molecules which react with amino acid residues and thus introduce carbonyl groups. Paraoxonase-1 (PON1) protects lipoproteins and membrane lipid from oxidative stress. Reaction of hydroxyl radicals (HO^·^) with guanine residues of DNA contributes to DNA oxidation. If not repaired, this oxidative damage can cause mutations and/or altered gene transcription.

ROS generated endogenously (mitochondria, metabolic process, inflammation, etc.) or from external sources modulate several biologic phenomena [12–15]. Under normal conditions, ROS and RNS are maintained by a balance due to enzymatic and nonenzymatic antioxidant defenses [12]. Other important sources of ROS are enzymes such as NADPH oxidases (NOXes), lipoxygenase (LOX), and myeloperoxidase (MPO) [17, 18]. Among these enzymes, special attention has been paid to NOXes, a family of enzyme complexes (NOX1–5 and Dual oxidases DUOX1/2). These membrane-bound enzymes generate ROS which are important for cellular signaling, development, apoptosis, and protection against pathogens [19]. Superoxide ions (O_2_^-·^) are generated by transferring electrons from *NADPH* inside the cell across the membrane. In addition to the superoxide-generating *NADPH* oxidase domain, DUOxs also have a peroxidase domain that converts the superoxide into hydrogen peroxide. MPO converts H_2_O_2_ to hypochlorous acid (HOCl), a strong oxidant that plays as a bactericidal agent in phagocytic cells. H_2_O_2_ is converted into a spontaneous reaction catalyzed by Fe^2+^ (Fenton reaction) to produce highly reactive hydroxyl radical (^·^OH) (Figure 2). Potential candidates for generation of oxidized lipoproteins in vivo are generated by NOX and MPO [20]. Other enzymes involved in ROS production are xanthine oxidase [21], *α*-ketoglutarate dehydrogenase complex [22], d-amino acid oxidases [23], and dihydrolipoamide dehydrogenase [24]. Inducible nitric oxide synthase (iNOS), an inducible protein, produces nitric oxide (NO) [25]. RNS such as peroxynitrite (ONOO^−^) are generated from NO and superoxide ions [25].

Protein amino acid residues can be modified by RNS, and 3-nitrotyrosine is a biochemical marker of oxidative damage to proteins [12, 26]. Protein modifications by oxidative stress result in the loss of their functions and may render proteins more prone to proteolytic degradation [26]. ROS and RNS can contribute also to oxidative damage to lipids and nucleic acids [27–29]. Lipid hydroperoxides, acrolein, malondialdehyde (MDA), and 4-hydroxy-2-nonenal (HNE) are useful biomarkers of oxidative stress to lipids [28, 29]. Therefore, in conditions characterized by an imbalance between ROS/RNS levels and antioxidants, oxidative cell injury may occur and trigger oxidation of lipids, proteins, and DNA. Many studies have demonstrated that cancer cells exhibit elevated ROS production [16]. Different molecular mechanisms are involved such as alterations of mitochondria and peroxisome, increased activity of metabolic transduction pathways, and transcriptional cellular receptor signaling [14–16]. Increased levels of ROS can sustain cellular proliferation and/or prolonged differentiation [14, 15]. In fact, cell redox potential affects transcription factors that regulate the expression of genes responsible for proliferation, apoptosis, angiogenesis, and production of cytokines [15].

## 3. ROS Generation, Oxidants, and Antioxidants in Bladder Cancer

### 3.1. NADPH Oxidase (NOX)

ROS production via NADPH oxidase (NOX) contributes to various types of cancer progression. As aforementioned, the NOX family comprises different isoforms: (NOX1–5). The pathobiological role of *NADPH* oxidase-mediated generation of ROS has also been studied in urothelial carcinoma (UC) of the urinary bladder [30]. Using immunohistochemistry, Shimada et al. [30] demonstrated that NOX4 was seldom expressed in normal urothelium. An overexpression was demonstrated in high-grade, superficially, and deeply invasive carcinomas but not in low-grade and noninvasive phenotypes. However, NOX4 expression level was not correlated to pathological parameters such as grade, stage, and tumor [30]. Furthermore, using human urothelial carcinoma cell lines in culture, it has been demonstrated that NOX4 silencing reduced ROS generation and suppressed cancer cell growth via p16-dependent cell cycle arrest at the G1 phase. These experimental evidences suggest that NOX4-mediated ROS generation can play a role in the molecular mechanisms involved in the early steps in urothelial carcinogenesis and cancer cell survival. In addition, NOX4-mediated enhancement of ROS generation resulted in more aggressive phenotype bladder cancer cells. These experimental evidences suggest that ROS generation via NOXes could represent a useful index in the cytological diagnosis of urothelial carcinoma (Table 1).

### 3.2. iNOS and Nitro-oxidative Stress

As aforementioned, iNOS is an inducible enzyme which produces NO [25]. Particularly, NO is an endogenous molecule that plays several physiological and pathophysiological roles in cancer biology and transmission of cellular signals [31]. The effect appears to be concentration-dependent. Low concentrations of NO (pico- to nanomolar range) lead to tumor promotion. On the contrary, proapoptotic functions leading to tumor suppression are triggered by higher NO concentrations (micromolar range) [31, 32]. Under oxidative stress and during inflammation, the cellular biosynthesis of iNOS is increased [33]. iNOS expression has been detected in bladder cancers as a higher expression, and activity has been observed in bladder tumoral tissue with respect to nontumoral tissues. The higher expression of *iNOS* is associated with an increased NO production [31]. An excessive production of iNOS also correlates with transition to more advanced stages of bladder cancer as demonstrated by Sandes et al. [31] (Table 1). The higher levels of NO observed in bladder cancer tissue and urine of BC patients with respect to healthy subjects are likely related to the higher activity of *iNOS* in tumoral tissue. Higher levels of NO concentrations have been also confirmed in serum [32, 33]. A potential role of NO in bladder cancer is supported by the comparison of serum concentrations of NO in BC patients before and after surgery. A decrease of NO levels has been observed after surgery with respect to the preoperative state. After surgery, NO levels did not differ statistically from concentrations in the control group [32]. Studies in cells in culture demonstrate that NO exerts a dichotomous effect in bladder cancer. Low concentrations are responsible for the modulation of the growth of bladder tumor cells [34]. High concentrations of NO in bladder cancer cells exposed to cytokine tumor necrosis factor and IL-1B trigger apoptosis [34].

## 4. Cellular and Extracellular Antioxidant Enzymes

The activity and expression of different antioxidant enzymes such as superoxide dismutase (SOD), catalase (CAT), glutathione peroxidase (GTPx), and paraoxonases (PONs) have been studied in BC patients. Literature data are summarized in Table 2.

### 4.1. Superoxide Dismutase (SOD)

Copper-zinc superoxide dismutase (Cu, Zn-SOD) plays a protective role in various types of tissue protecting them from oxidative damage. Lower levels of SOD activity in the tumor tissue of bladder cancer patients in comparison to benign tumors have been observed [35–38]. SOD expression is significantly lower in invasive transitional cell carcinomas than in superficial transitional cell carcinomas [35]. Modifications of SOD have been demonstrated also in serum isolated from BC patients [39, 40] (Table 2).

### 4.2. Catalase (CAT)

CAT protects cells against the excessive formation of reactive oxygen species and prevents the accumulation of H_2_O_2_. A decreased CAT expression [35] and activity [36, 38] in cancerous bladder tissue comparison with control bladder tissues have been reported. Modifications of CAT activity were observed also in serum of BC patients [39].

### 4.3. Glutathione and Peroxidase (GTPx)

GSH exerts a key antioxidant intracellular role [41] and is also involved in many metabolic processes. GSH and the enzymes involved in its metabolism such as glutathione S-transferase, glutathione peroxidase, and glutathione reductase play an important role in several diseases, including cancer [41]. Lower values of GTPx activity were found in erythrocytes and in bladder cancer tissues, both in comparison with the bladder tissues of patients without tumors, and in comparison with normal tissues of the bladders with tumors [37, 42, 43]. Therefore, a decrease of GSH could contribute to a shift in an intracellular environment to a prooxidant state leading to multiple changes.

### 4.4. Paraoxonase 1 (PON1)

Among antioxidant and anti-inflammatory enzymes, paraoxonase includes three different proteins such PON1, PON2, and PON3. All enzymes behave as an antioxidant [13, 44–46]. Mainly, PON1 and PON3 are localized in the plasma. PON2 is localized in the plasma membrane, endoplasmic reticulum, nuclear envelop, and inner mitochondrial membrane. These enzymes protect biological membranes and lipoproteins against potentially harmful ROS which contribute to lipid peroxidation (Figure 2). Many studies have investigated the relationship between PON enzymes and various diseases that involve oxidative stress [13, 44–46]. PON1 activities could be evaluated using different substrates including paraoxon (PON1 paraoxonase activity) and phenyl-acetate (PON1 arylesterase activity). PON1 paraoxonase and arylesterase activities were significantly decreased in the serum of BC subjects with respect to controls [47].

More recently, also, a decrease of serum paraoxonase-1 concentration has been demonstrated in patients with urinary bladder cancer. The lower serum PON1 concentrations were associated with higher levels of chemokine (C-C motif) ligand 2 C-reactive protein than the control individuals [48]. Moreover, a relationship between *PON1* and clinical data was reported with lower PON1 concentration in patients with tumor recurrence with respect to patients without tumor recurrence [48]. Several studies in chronic diseases have demonstrated that a low PON1 activity in serum exposes subjects to a higher oxidative stress [49]. The relationship between PON1 gene polymorphism and BC has demonstrated that RR genotype was more common in bladder tumors [50].

### 4.5. Paraoxonase 2 (PON2)

PON2 is a member of multiple gene family of paraoxonase that represents an intracellular enzyme localized in the plasma membrane, endoplasmic reticulum, nuclear envelope, and inner mitochondrial membrane. A protective effect against lipid peroxidation and intracellular ROS formation is exerted by PON2 [44]. Due to its localization in the ER and mitochondria, PON2 could act as an antiapoptotic effect that can be of physiopathological relevance in tumor cells [43–46]. In addition to alterations of activity and concentration of PON1, modifications of PON2 have been demonstrated in BC [44, 51]. The comparison of expression levels of PON2 in paired tumor and normal bladder tissue samples from patients affected with BC, most of which underwent radical cystectomy for the treatment of advanced disease (pT3-4), has shown that PON2 expression levels were significantly higher (2.01-fold) in BC compared with those detected in normal tissue [51]. Furthermore, PON2 expression in urinary exfoliated cells obtained in BC patients was significantly higher compared to that in patients affected with tumors invading subepithelial connective tissue or extending outside the bladder (pT1-3). PON2 overexpression on bladder tumor cells (T24) was associated with higher proliferation and lower susceptibility to oxidative stress by tert-butyl hydroperoxide. Upregulated levels of *PON2* have been detected in different types of cancer cells including BC, and a possible involvement of the role of *PON2* higher expression in apoptotic escape of tumor cells has been suggested [51].

## 5. Markers of Lipid, Protein, and Nucleic Acid Oxidative Stress in Bladder Cancer

Previous studies have demonstrated higher levels of biochemical markers of oxidative stress of lipid, proteins, and nucleic acids in BC patients (Table 3).

### 5.1. Lipid Peroxidation

A significant increase of malondialdehyde (MDA) has been observed by different authors in the serum and plasma of BC patients [33, 40, 43]. The higher levels of MDA, the major aldehyde product of lipid peroxidation of membrane polyunsaturated fatty acids by free radicals, demonstrates that oxidative stress realizes in biological membranes and/or plasma lipoproteins in bladder cancer patients. Badjatia et al. confirmed an increase of lipid peroxidation in more advanced bladder cancer [40].

Other markers of lipid peroxidation have been studied in bladder cancer tissue. Higher levels of 4-HNE were observed in the bladder cancer tissues compared with the non tumorous tissue. 4-HNE was mainly detectable in the cytoplasm of cancer cells [52]. Even acrolein is a byproduct of lipid peroxidation [53]. Higher levels of *γ*-OH-acrolein-dG DNA adducts have been reported in bladder tumor tissues compared to normal human urothelial mucosa. It has been suggested that tumor cells could be more susceptible to adduct formation and/or tumor cells have a lower repair capacity. Whatever are the causes of the higher levels of acrolein in the bladder tumor cells than in normal human urothelial mucosa, the increased levels may favour the formation of higher levels of acrolein-dG DNA adducts [54]. Acrolein adduction of DNA, if not repaired efficiently, has the potential cause to critical gene mutations, suggesting that acrolein may be mutagenic and may contribute to the process of carcinogenesis.

A deregulation of oxidative/antioxidant balance in bladder cancer is also suggested by the significant modifications of total antioxidant status (TAS) and total oxidant status (TOS) in the serum of BC subjects. The TAS indicates the overall antioxidative status of the serum, while the TOS denotes the oxidative status of the serum [33, 40, 47]. The significant decrease of the oxidative stress index (OSI) calculated as the ratio between TOS and TAS values confirms that BC is associated with oxidative stress [47].

### 5.2. Protein Oxidation

ROS and RNS favour oxidative stress of cell proteins. Levels of protein carbonyl groups are remarkably higher in bladder cancer patients than in healthy controls [55]. Patients with bladder cancer have also significantly lower levels of total thiol groups and protein-bound thiol groups as compared to healthy controls [55].

### 5.3. Nucleic Acids

RNS like ROS can directly damage the molecule DNA, while inhibiting its repair. The formation of 8-hydroxy-2′-deoxyguanosine (8-OHdG), oxidative damage to guanine (G), is the most common form of oxidative DNA damage [29, 56]. This base modification increases by 35–50% in individuals using tobacco smoke, a well-known carcinogenic source of ROS [57]. Higher levels of 8-OHdG were found in bladder cancer tissues than in the surrounding cancer-free tissues at various stages of the disease [58]. All these results confirm that oxidative stress realizes in BC. Oxidative stress reflects also in mitochondrial DNA (mtDNA) damage mutations and mtDNA instability as demonstrated in human cancers [59]. The increased levels of mtDNA found in both the urine and plasma of patients with bladder cancer may suggest endogenous tumor necrosis and release of mtDNA into both the urine and plasma [60].

## 6. Conclusions

The production of ROS and RNS through either endogenous or exogenous insults plays a major role in the aging process and age-related disease. Numerous epidemiological, experimental, and clinical studies have demonstrated that markers of oxidative stress are associated with the development and progression of cancer. The higher levels of markers of lipid, protein, and DNA oxidation demonstrated in BC tissues confirm a potential role of oxidative stress in the molecular mechanism of the disease. Literature data suggest an overproduction of NO and/or a deficiency in the antioxidant systems (SOD, CAT, and GTPx) in the bladder tissue, serum, and plasma of BC patients. Among antioxidant enzymes, a decrease of serum PON1 has also been reported. Several molecular mechanisms could be implicated in the cancer-related decrease in the activities of antioxidant enzymes such as a downregulation of synthesis by proinflammatory cytokines such as TNF*α* and IL-1. Overall, the results of our review confirm that bladder cancer is associated with a shift in the antioxidant/pro-oxidant balance. The cause of the imbalance is unknown. Dietary and environmental factors, prolonged exposure to carcinogens, and/or accumulation of genetic and cellular damage could be the most powerful candidates.

## Figures and Tables

**Table 1:** Modifications of expression of NADPH oxidase (NOX4), inducible nitric oxide synthase (iNOS), and nitric oxide (NO) levels in bladder cancer patients and controls.

Markers	Samples	Levels	References
NADPH oxidase (NOX4)	Tissue BC tissue (*n* = 82) vs. normal bladder tissue (*n* = 82)	2-fold increase in NO4-positive cells (/1000) (*p* < 0.01)	[30]

Inducible nitric oxide synthase (iNOS)	Tissue Invasive BC (*n* = 14) vs. noninvasive BC (*n* = 31) High grade BC (*n* = 33) vs. low grade BC (*n* = 12)	iNOS pos (%) 100% vs. 71% (*p* = 0.0399) 91% vs. 50% (*p* = 0.0062)	[31]

Nitric oxide (NO) levels	Tissue BC tissues (*n* = 20) vs. normal bladder tissue (*n* = 15)	NO levels (nmol/g tissue) 36.9 ± 4.37 vs. 24.65 ± 2.7 (*p* < 0.0001)	[32]
	Serum BC patients (*n* = 20) vs. controls (*n* = 41)	NO levels (*μ*mol/L) 61.25 ± 4.95 vs. 26.61 ± 6.13 (*p* < 0.0001)	[33]
	BC patients (*n* = 35) vs. controls (*n* = 32)	17.1 ± 1.4 vs. 8.1 ± 0.8 (*p* < 0.05)	[32]
	Urine BC patients (*n* = 20) vs. controls (*n* = 41)	NO levels (*μ*mol/100 mg urinary creatinine) 4.36 ± 0.34 vs. 1.69 ± 0.31 (*p* < 0.0001)	[32]

**Table 2:** Modifications of antioxidant enzymes in bladder cancer patients and controls.

Biochemical markers	Samples	Enzyme activities and levels	References
Superoxide dismutase (SOD)	Tissue BC tissue (*n* = 75) vs. normal bladder tissue (*n* = 30)	SOD positive (%) 49.3% vs. 80% (*p* = 0.007)	[35]
	BC tissue (*n* = 25) vs. normal bladder tissue (*n* = 15)	SOD activity (IU/mg) 0.0715 ± 0.0056 vs. 0.1407 ± 0.0134 (*p* < 0.0001)	[36]
	BC tissues (*n* = 25) vs. normal bladder tissue (*n* = 26)	SOD activity (U/mg) 40 vs. 80	[37]
	BC tissue (*n* = 36) vs. normal bladder tissue (*n* = 9)	SOD activity (U/mg prot) 16.5 ± 4.5 vs. 1.49 ± 0.61 (*p* < 0.05)	[38]
	Serum BC patients (*n* = 50) vs. controls (*n* = 50)	SOD activity (U/mL) 149.14 ± 29.65 vs. 201 ± 31.4 (*p* < 0.001)	[39]
	BC patients (*n* = 50) vs. controls (*n* = 40)	28.49 ± 14.03 vs. 194.0 ± 28.48 (*p* < 0.001)	[40]

Catalase (CAT)	Tissue BC tissue (*n* = 75) vs. normal bladder tissue (*n* = 30)	CAT positive (%) 44% vs. 73.3 % (*p* = 0.012)	[35]
	BC tissue (*n* = 25) vs. normal bladder tissue (*n* = 15)	CAT activity (IU/mg) 6.220 ± 0.991 vs. 11.651 ± 3.684 (*p* < 0.0001)	[36]
	BC tissue (*n* = 36) vs. normal bladder tissue (*n* = 9)	CAT activity (IU/mg) 33.7 ± 11.5 vs. 60.2 ± 26.9 (*p* < 0.01)	[38]
	Serum BC patients (*n* = 50) vs. controls (*n* = 50)	CAT activity (u/L) 10.4 ± 2.4 vs. 20 ± 4.3 (*p* < 0.001)	[39]

Glutathione peroxide (GTPx)	Tissue BC tissue (*n* = 75) vs. normal bladder tissue (*n* = 30)	GTPx positive (%) 45.3% vs. 63.3 % (*p* = 0.146)	[35]
	BC tissue (*n* = 25) vs. normal bladder tissue (*n* = 15)	GTPx activity (IU/mg) 0.232 ± 0.009 vs. 0.523 ± 0.034 (*p* < 0.0001)	[36]
	BC tissue (*n* = 25) vs. normal bladder tissue (*n* = 26)	GTPx activity (U/g) 35 vs. 85	[37]
	Serum BC patients (*n* = 50) vs. controls (*n* = 50)	GTPx activity (U/L) 131.00 ± 14.46 vs. 170 ± 28 (*p* < 0.001)	[39]
	BC patients (*n* = 50) vs. controls (*n* = 40)	1693.09 ± 544.01 vs. 6906 ± 847 (*p* < 0.001)	[40]
	Erythrocyte Grade III BC patients (*n* = 22) vs. controls (*n* = 23)	GTPx activity (U/g Hb) 4 vs. 5 (*p* < 0.001)	[43]

Glutathione (GSH)	Tissue BC tissue (*n* = 7) vs. normal bladder tissue (*n* = 14)	GSH levels (*μ*M/mg) 1.345 ± 1.252 vs. 7.887 ± 6.176 (*p* < 0.001)	[42]
	BC tissues (*n* = 25) vs. normal bladder tissue (*n* = 26)	GSH levels (mg/mL) 35 vs. 75	[37]

Paraoxonase-1 (PON1)	Serum BC patients (*n* = 56) vs. controls (*n* = 57)	PON1 paraoxonase activity (U/L) 103.35 ± 41.44 vs. 137.63 ± 53.37 (*p* = 0.0001) PON1 arylesterase activity (U/L) 131.83 ± 39.94 vs. 168.82 ± 37.34 (*p* = 0.0001)	[47]
	BC patients (*n* = 29) vs. controls (*n* = 61)	PON1 paraoxonase activity (U/L) 239.1 (116.6–457.4) vs. 253.1 (149.5–434.7)	[48]
	BC patients (*n* = 29) vs. controls (*n* = 61)	PON1 concentration (mg/L) 70.6 (18.2–185.2) vs. 101.6 (52.9–325.1)	

Paraoxonase-2 (PON2)	Tissue BC tissues (*n* = 17) vs. normal bladder tissue (*n* = 17)	PON2 expression levels: 2.01-fold higher in BC tissue vs. normal tissue (*p* < 0.05)	[49]
	BC tissues vs. normal bladder tissue	PON2 expression levels: 4.01-fold higher BC tissue vs. normal tissue (*p* < 0.05)	[44]

**Table 3:** Markers of oxidative stress in bladder cancer patients and control subjects.

Biochemical markers	Samples	Levels	References
Malondialdehyde (MDA)	Serum BC patients (*n* = 50) vs. controls (*n* = 40)	MDA (nmol/mL) 13.91 ± 8.59 vs. 2.12 ± 0.78 (*p* < 0.0001)	[40]
	BC patients (*n* = 35) vs. controls (*n* = 32)	16.8 ± 1.6 vs. 9.1 ± 0.4 (*p* < 0.05)	[33]
	Grade III BC patients (*n* = 22) vs. controls (*n* = 23)	MDA (nmol/mL) 4 vs. 1 (*p* < 0.001)	[43]

Acrolein-dG DNA adducts	Tissue BC tissue (*n* = 10) vs. normal bladder tissue (*n* = 19)	DNA adduct/dG × 10^7^ (63 ± 25) × 10^−7^/dG vs. (25 ± 10) × 10^−7^/dG	[54]

Total antioxidant status (TAS)	Serum BC patients (*n* = 56) vs. controls (*n* = 57)	TAS (mmol Trolox equiv./L) 0.91 ± 0.17 vs. 0.99 ± 0.12 (*p* < 0.010)	[47]
	BC patients (*n* = 50) vs. controls (*n* = 40)	TAS (mM) 0.99 ± 0.06 vs. 1.45 ± 0.22 (*p* < 0.001)	[40]
	BC patients (*n* = 35) vs. controls (*n* = 32)	TAS (mmol Trolox equiv./L) 1.1 ± 0.1 vs. 2.5 ± 0.2 (*p* < 0.05)	[33]

Total oxidant status	Serum BC patients (*n* = 56) vs. controls (*n* = 57)	TOS (mmol H_2_O_2_ equiv./L) 24.68 ± 6.84 vs. 17.55 ± 7.79 (*p* = 0.001)	[47]

Protein carbonyl groups (PCO)	Plasma BC patients (*n* = 43) vs. controls (*n* = 28)	PCO (nmol/mg protein) 0.682 ± 0.094 vs. 0.606 ± 0.077 (*p* < 0.001)	[55]

Protein thiol	BC patients (*n* = 43) vs. controls (*n* = 28)	Protein thiol (*μ*mol/L) 311.427 ± 89.507 vs. 366.181 ± 57.717 (*p* < 0.01)	

8-OHdG	Tissue BC tissue (*n* = 31) vs. normal bladder tissue (*n* = 31)	8-OhdG ng/mL/mg DNA 72.7 ± 16.6 vs. 42.2 ± 15.3 (*p* = 0.072)	[58]

## Abbreviations

ARE: Arylesterase
CAT: Catalase
DUOX: Dual oxidase
ER: Endoplasmic reticulum
GSH: Glutathione
GTPx: Glutathione peroxidase
4-HNE: 4-Hydroxynonenal
iNOS: Inducible NO synthase
IL-1: Interleukin-1
LOX: Lipoxygenase
MDA: Malondialdehyde
MPO: Myeloperoxidase
NOXes: NADPH oxidases
OSI: Oxidative stress index
PON: Paraoxonase
PON1: Paraoxonase-1
PON2: Paraoxonase-2
PON3: Paraoxonase-3
PHS: Prostaglandin H synthase
RNS: Reactive nitrogen species
ROS: Reactive oxygen species
SOD: Superoxide dismutase
TAS: Total antioxidant system
TOS: Total oxidant system
TNF*α*: Tumor necrosis factor-alpha.
